# Risk of Iodine Deficiency in Extremely Low Gestational Age Newborns on Parenteral Nutrition

**DOI:** 10.3390/nu12061636

**Published:** 2020-06-01

**Authors:** Neelakanta Kanike, Sharon Groh-Wargo, Megan Thomas, Edward K. Chien, Maroun Mhanna, Deepak Kumar, Sarah Worley, Ravinder J. Singh, Prem S. Shekhawat

**Affiliations:** 1 Department of Pediatrics, Division of Neonatology, Case Western Reserve University, Metro Health Medical Center, Rm C.G75, 2500 MetroHealth Drive, Cleveland, OH 44109, USA; neelkanike@gmail.com (N.K.); sgrohwargo@metrohealth.org (S.G.-W.); mmhanna@metrohealth.org (M.M.); dkumar@metrohealth.org (D.K.); 2 Department of Obstetrics & Gynecology, Case Western Reserve University, Metro Health Medical Center, Cleveland, OH 44109, USA; meganthomas320@gmail.com (M.T.); chiene@ccf.org (E.K.C.); 3 Department of Quantitative Health Sciences, Section of Biostatistics, Cleveland Clinic, Cleveland, OH 44195, USA; worleys@ccf.org; 4 Department of Laboratory Medicine & Pathology, Mayo Clinic Foundation, Rochester, MN 55905, USA; singh.ravinder@mayo.edu

**Keywords:** neonatal iodine deficiency, subclinical hypothyroidism, extremely low gestation age neonates, total parenteral nutrition, maternal iodine deficiency

## Abstract

**Iodine** is an essential component of thyroid hormones, which play a critical role in neurodevelopment. The iodine status of pregnant women and their newborns is not checked routinely. Extremely Low Gestational Age Newborns do not receive Iodine supplementation while on parenteral nutrition (PN). We measured urine iodine levels and thyroid function tests in 50 mother–infant dyads at birth, at 1 week, 1, 2, 3 months and near discharge. We correlated maternal and neonatal urine iodine levels with thyroid functions and measured iodine levels in milk and PN. In our study, 64% of mothers were iodine deficient at the time of delivery, their free T4 levels were 0.48 (0.41–0.54) ng/dL with normal thyroid-stimulating hormone (TSH). Iodine levels were thirty-fold higher in extremely low gestational age newborns (ELGAN) exposed to iodine comparing to full terms (*p* < 0.001), but this effect lasted <1 week. At 1 month of age, ELGAN on PN developed iodine deficiency (*p* = 0.017) and had high thyroglobulin levels of 187 (156–271) ng/mL. Iodine levels improved with enteral feeds by 2 months of age (*p* = 0.01). Iodine deficiency is prevalent among pregnant women and ELGAN; in particular, those on PN are at risk of hypothyroidism. Iodine supplementation during pregnancy and postnatally should be considered to avoid iodine deficiency.

## 1. Introduction

Iodine is an essential component of thyroid hormones, thyroxine (T4) and triiodothyronine (T3), which are critical for central nervous system development, including neuronal migration and myelination during fetal and early postnatal life. Though iodine deficiency can affect people of any age, pregnant women and children are the most vulnerable high risk groups [1]. The requirement of iodine increases by 50% during pregnancy due to the increased production of maternal thyroxine [2]. In pregnant women, iodine deficiency increases the risk of major neurodevelopmental deficits and growth retardation in the fetus, as well as miscarriage and stillbirth [3]. Chronic, severe iodine deficiency in-utero causes cretinism, a condition characterized by intellectual disability, hearing loss, mutism, motor spasticity, stunted growth, delayed sexual maturation, and other physical and neurological abnormalities [3]. Less severe iodine deficiency during pregnancy and early postnatal life is also associated with neurodevelopmental deficits in infants and children [4,5,6]. Mild to moderate maternal iodine deficiency has also been associated with an increased risk for attention deficit hyperactivity disorder in children [7].

While iodine deficiency in the general population is a common cause of hypothyroidism worldwide, it is less common in developed countries like the United States. But there is increasing evidence that pregnant women in developed countries are mildly to moderately iodine deficient, particularly those not taking iodine-containing prenatal vitamin supplements [8]. The National Health and Nutrition Examination 2005–2008 survey in the USA demonstrated that 57% of pregnant women had iodine deficiency, defined as urinary iodine concentration (UIC) <150 mcg/L [9]. The National Children’s Study from 2009 with 501 pregnant women reported iodine deficiency in 45.3% of the participants [10]. Dairy, grain, seafood and iodized salt are the major sources of iodine in the United States. Nevertheless, there is wide variation in iodine in foods and iodine content is seldom included in nutritional labeling [11]. Reductions in the US dietary iodine level have been variously ascribed to reduction in the iodine content of dairy products, the removal of iodate dough conditioners in commercially produced bread, new recommendations for reduced salt intake for blood pressure control, and the increasing use of non-iodized salt in manufactured or premade convenience foods [12].

Preterm infants are at highest risk for developing iodine deficiency. They are deprived of the maternal supply of thyroid hormones and iodine, before their own thyroid gland has been able to accumulate as much iodine as in term newborns. Moreover, infants receiving total parenteral nutrition (TPN) for long duration like extreme preterm babies have very restricted iodine exposure [13,14]. Preterm infants have low circulating levels of T4 and free T4; the degree of hypothyroxinemia is greater in extremely low gestational age newborns (ELGAN) [15]. For years, their alterations in the circulating thyroid hormone levels have been considered an expression of their physiological hypophyseal-pituitary thyroid immaturity (euthyroid sick syndrome) or a manifestation of nonthyroidal illness, but follow-up studies of 640 children born preterm up to 5 and 9 years of age [16] and another 400 preterm infants followed up to 2 year of age [4] have confirmed that severe hypothyroxinemia plays an significant causative role in their frequently impaired mental development and sometimes disabling cerebral palsy [4,16].

Iodine deficiency contributes to about 30% of the hypothyroxinemia in enterally fed preterm infants of 27–30 weeks gestational age (GA) [17]. Iodine deficiency is most commonly assessed by measuring UIC because approximately 90% of dietary iodine is excreted in the urine [18]. Studies in healthy preterm infants have suggested that enteral iodine intakes of ≥30 mcg/kg/d are required to remain in positive balance [17,19]. Current recommendation for enteral intakes in preterm infants is 11–55 mcg/kg/day [20]. Preterm infant formulas contain 20–170 mcg/L iodine, mother’s own human milk contains 50–150 mcg/L iodine, and donor human milk contains 33–117.5 mcg/L iodine [21,22]. Even with human milk fortification using commercially available US products, iodine content remains below intake recommendations for preterm infants [21]. 

To avoid the risk of iodine deficiency in preterm infants who are dependent on TPN, an intake of 1 mcg/kg/d is recommended [14,19,23]. This recommendation takes into account the potential for significant absorption through the skin from using iodine-containing antiseptics. However, the skin cleansing of preterm infants has been moving away from this practice to using 2% chlorhexidine. Recent studies show that doses of 1 to 3 mcg/kg/day iodine included in typical TPN formulations is not sufficient for adequate iodine status [13,24]. Hypothyroidism as a result of iodine deficiency in a TPN-dependent preterm infants with short bowel syndrome has been well described [25,26]. Currently, the neonatal parenteral multi–trace mineral product manufactured in the United States do not contain iodine and no IV single nutrient iodine product is available. Thus, there is risk for iodine deficiency and parenteral supplementation requirements need to be reviewed. Considering the above evidence, we hypothesized that women delivering in our urban inner-city Cleveland, Ohio hospital are iodine deficient, and that infants born to these women, especially premature infants, are likely to be iodine deficient, and that low iodine stores may affect their thyroid function.

## 2. Objectives 

Our primary aim was to assess the iodine status of mother–infant dyads at the time of delivery. Our secondary aim was to assess neonatal iodine and thyroid function status longitudinally from birth till 40 weeks post conception age or prior to discharge and compare iodine levels by gestational age groups.

## 3. Study Design/Methods

**Figure 1:**
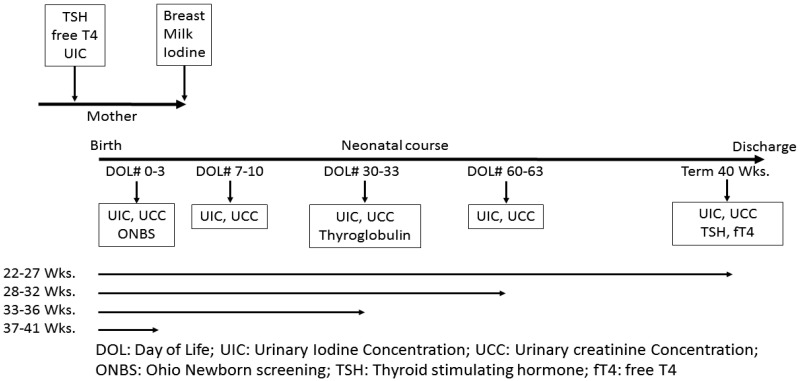
Flow diagram showing timing of laboratory assays in various gestational age subjects.

This prospective observational longitudinal study was conducted at MetroHealth Medical Center, Cleveland, Ohio between 1 February 2016 to 31 January 2017. The study was approved by MetroHealth Medical Center Institutional Review Board. Women delivering between 22–41 weeks gestation were included in the study following written consent. Newborn infants born to these women were evenly divided into four groups by gestational age (GA): Group 1: 22 to 27 weeks (extremely low gestational age newborns); Group 2: 28 to 32 weeks (low gestational age newborns); Group 3: 33 to 36 weeks (late preterm newborns); and Group 4: 37 to 41 weeks (term newborns). Infant subjects were followed until hospital discharge or until they reached 40 weeks post conceptional age, whichever came first. Pregnant women were eligible if they had no history of thyroid disease, or treatment with anti-thyroid medications like propylthiouracil, methimazole and amiodarone. Infants were excluded if they had a known major congenital malformation or one was diagnosed after birth. To assess maternal iodine status, urine was collected during labor after enrollment for urine iodine and creatinine concentrations. Spot urine sample was collected before the application of betadine to perineal area for consistency. Similarly, blood was collected to assess thyroid-stimulating hormone (TSH) and free thyroxine (fT4) (Figure 1). 

To estimate the iodine status of newborns, urine was collected at different time periods using a urine bag as part of standard of care or in extremely preterm infants using cotton balls: during the first 3 days of life, at 1-week post-natal age (PNA), at 1-month PNA, then monthly through 40 weeks corrected GA and prior to discharge (Figure 1). Urine was collected for these tests by applying a urine bag or in extremely preterm infants using cotton balls. Samples were snap frozen at −20 °C until assayed at Mayo Clinic, Rochester, MN. Iodine levels in urine, breast milk, intravenous fat emulsion and parenteral nutrition samples were analyzed by inductively coupled plasma-mass spectrometry (ICP-MS) in standard mode [27]. The lowest detection rate for UIC assay was 10 μg/L, and samples reported as <10 μg/L were analyzed as 5 μg/L.

Neonatal and infant thyroid status was assayed at the newborn screen, 30 days and at 40 weeks corrected GA (Figure 1). The first TSH level measured by the immunofluorometric method was available from our state of Ohio’s newborn screen (ONBS) [28]. Low TSH values when reported by ONBS as <2.91 mIU/mL, were as assigned a value of 2.0 mIU/mL for analysis. The second TSH level and fT4 were obtained prior to discharge as a part of routine clinical practice in our neonatal intensive care unit. Thyroglobulin is a sensitive indicator of low iodine intake and it is also an early marker of iodine repletion [29]. A total of 1 ml of blood was drawn for thyroglobulin levels around 1 month of age. Thyroid function tests were performed by enzyme-linked immunosorbent assay method.

To assess nutritional iodine content, we measured the iodine content in maternal breast milk and in random donor milk samples used for enrolled infants, PN samples, and fat emulsion samples. Maternal breast milk was assayed in the first week of life after establishment of lactation. Maternal breastmilk samples were assayed by pooling pumped breast milk over a 24-h period. This was performed for two different 24-h periods. For babies who were fed donor breast milk, we collected two 1-ml samples of donor breast milk. We also analyzed 10 random TPN samples and 5 intravenous fat emulsion samples for iodine content.

Maternal demographic data collected included age, race, use of prenatal vitamins and type of salt intake and mode of delivery and use of topical iodine for procedures. Infant data included GA, gender, birth weight, umbilical line placement, type of nutrition, for example, maternal breast milk or donor breast milk or formula, and number of days on TPN.

## 4. Statistical Methods

Data were described using medians and quartiles for continuous variables and counts and percentages for categorical variables. The correlation between maternal and newborn iodine levels, and iodine/creatinine ratios were assessed using Spearman rank correlation coefficients. Gestational age groups were compared on maternal and newborn iodine levels at birth and throughout follow-up using Kruskal–Wallis tests with the Steel–Dwass multiple comparison procedure. Sample sizes for individual variables reflect missing data. All analyses were performed on a complete-case basis. All tests were two-tailed and performed at a significance level of 0.05. SAS 9.4 software (SAS Institute, Cary, NC) was used for all analyses and graphs. 

## 5. Results

Fifty-two mother–infant dyads were enrolled in the study of which one mother and one infant had extremely high UIC, thought to be due to assay error, and so were excluded from analysis, and one infant had incomplete data due to transfer to another facility thus 50 subjects were included in the final data analysis. Table 1 summarizes infant demographic characteristics for the cohort. Our cohort of women were mostly Caucasians and African Americans. Table 2 summarizes maternal and neonatal clinical characteristics. Sixty-four percent of mothers were iodine deficient at the time of delivery with median iodine level of 98 mcg/L (normal > 150 mcg/L). Their fT4 levels were low at 0.48 ng/dL (normal 0.86–1.90 ng/dL) with normal TSH values, suggestive of subclinical hypothyroxinemia. In the newborns, urinary iodine excretion was much higher at lower GA than term. The type of delivery was not significantly associated with newborn’s first UIC. In the full cohort, GA had a strong negative correlation with newborn iodine levels at birth and first TSH levels in newborns negatively correlated with first UIC (*p* < 0.001). The first UIC was significantly higher in newborns with umbilical lines compared to those who did not (*p* < 0.001).

**Figure 2:**
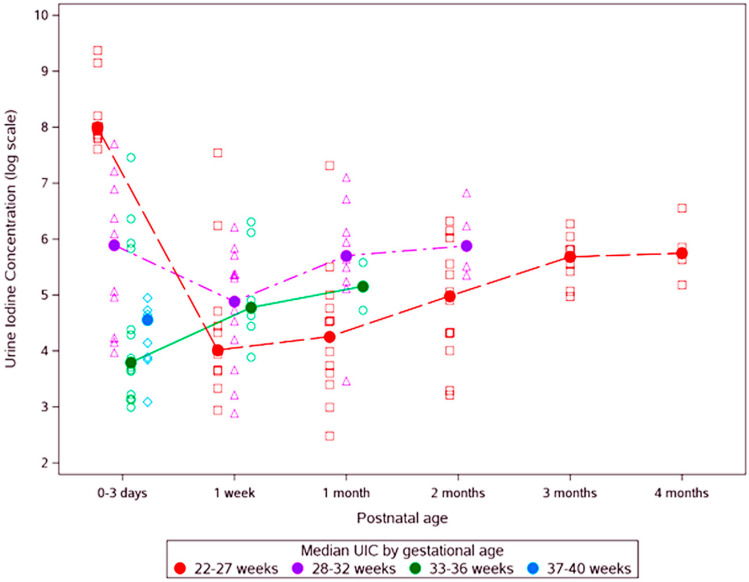
Changes in neonatal urinary Iodine levels (log scale) over time in various gestational age groups.

Table 3 compares iodine levels at birth and over time across the four GA groups. The groups did not differ significantly on maternal iodine variables. Newborn iodine variables were highest in the most preterm group at birth; however, by the third UIC measurement, neonates in the GA 22–27 weeks group had significantly lower UIC than neonates of GA 28–32 weeks. The UIC levels were thirtyfold higher in ELGAN after the application of iodine containing antiseptic for umbilical line placement procedures (*p* < 0.01), but this effect lasted less than one week. ELGAN on TPN had iodine deficiency with a median UIC of 73 [34, 133) mcg/L compared to others (*p* = 0.017). Infants who remained admitted to the NICU at 1 month of age had high median thyroglobulin levels of 187 [156, 271) ng/mL (normal 13–47 ng/mL) suggestive of subclinical hypothyroidism. These levels improved after instituting enteral nutrition by two months of age in ELGANs and their TSH and fT4 levels were also in the normal range prior to discharge. Figure 2 shows newborn UIC values over time, by gestational age groups. The trajectory of UIC of the smallest GA group, starting high, falling below other groups, and then slowly rising again by 2-month age was typical in contrast to term or near-term neonates who had borderline normal but steady UIC. 

The iodine content in the 13 breast milk samples ranged from 5 to 1924 mcg/L, with a median of 66 mcg/L. Donor milk also had varied levels of iodine in samples from different lots, ranging from 86 to 122 mcg/L. There was no detectable iodine in any of the TPN samples and five lipid emulsion samples were found to have iodine content in the range of 33 to 34 mcg/L. 

## 6. Discussion

According to the WHO/International Council for the Control of Iodine Deficiency Disorders/UNICEF criteria, median UICs, <150 and ≥150 mcg/L for pregnant women, and median UICs, <100 and ≥100 mcg/L for postpartum mothers, are considered as iodine deficiency and sufficiency, respectively [30]. The median UIC has been widely used as a biomarker of population iodine intake and deficiency [18]. Our study has demonstrated that iodine deficiency is common in our urban population of pregnant women who consumed low iodine diets and did not receive iodine supplementation during pregnancy. We found 64% of mothers in our population were iodine deficient, even though most of them (88%] were receiving prenatal vitamins, which usually did not contain iodine. They showed biochemical evidence of subclinical hypothyroidism, as demonstrated by low UIC on spot urine checks, and are at risk for subclinical hypothyroxinemia. ELGAN born to these mothers had high UIC after topical application of iodine but this effect did not last beyond the first week of life. ELGAN infants on TPN developed low iodine levels by one month of age and showed biochemical evidence of hypothyroidism but this situation improved after introduction of enteral feeds. 

Iodine deficiency has been historically described as a public health problem in metro Cleveland and northeast Ohio region [31]. The Institute of Medicine recommends a daily iodine intake of 220 mcg during pregnancy and 290 mcg during lactation [32]. The World Health Organization recommends 250 mcg of iodine daily for pregnant and lactating women [33]. The American Thyroid Association has recommended that women take prenatal vitamins that contains 150 mcg iodine in the form of potassium iodide daily during pregnancy and lactation [34,35]. The iodine content of prenatal multivitamins, however, is not mandated in the United States. There is wide variation in the iodine content of the prenatal vitamins available in the market. Of the 223 types of prenatal multivitamins available in the United States, only 51% contain any iodine [36]. Iodine in US prenatal multivitamins is typically derived either from potassium iodide (KI] or from kelp. The iodine content in prenatal multivitamin brands containing kelp may be inconsistent due to variability in kelp iodine content [37,38]. Iodine supplementation in pregnant women improves maternal thyroid indices and may benefit aspects of cognitive function in children, even in marginally iodine-deficient areas [39].

Iodine deficiency in its most extreme form, results in cretinism, but of much greater public health importance are the subtler degrees of brain damage and reduced cognitive capacity that affects the entire population [6]. Neonates and especially preterm infants are a very vulnerable population at risk of suffering the consequences of both iodine deficiency because of the impact of neonatal hypothyroxinemia on brain development. Enteral and TPN solutions are the principal potential sources of iodine intake in these infants [40]. Our study has shown that TPN does not supply the premature neonate any iodine, and lipid emulsions provide negligible amounts of iodine. Therefore, iodine supplements should be added while the babies are on TPN. Although there is a recommendation that a minimum of 1 mcg/kg/day of iodine be supplemented in TPN given to newborns, currently, there is no IV source available in the US [14,19].

The iodine content of human milk can vary depending on maternal iodine status and dietary intake as well as geographic location. Colostrum contains the greatest amount of iodine, with concentrations as high as 200–400 mcg/L. [41] Iodine content then decreases in mature human milk to generally between 50 and 150 mcg/L [21,42]. Our results also demonstrate substantial variability in the iodine content of breast milk and donor breast milk. Irrespective of wide variation in iodine content in breast milk or formula, the babies in our study were able to reach iodine sufficiency by 2 months of age, when the enteral intake was around 140 mL/kg/day, which provided sufficient iodine intake.

The high UIC levels in low GA infants reflect their exposure to iodine containing antiseptic as the samples were collected after the exposure. All ELGAN babies get umbilical lines in the first hours after birth, so it was practically impossible to get first urine samples before this exposure. Consequently, we could not assess the correlation between maternal and infant iodine status at birth. Exposure to topical iodine (10,000 mcg of iodine/mL] puts these infants to risk for iodine excess and hypothyroxinemia due to Wolff-Chaikoff effect [43]. Toxic levels of iodine were seen in our ELGAN group with median UIC level of 2954 mcg/L, after the application of iodine containing antiseptics for procedures. Although the initial UIC levels were very high in ELGAN, it lasted less than one week and then levels gradually returned to normal range. Extremely preterm infants on TPN were more likely to develop iodine deficiency and had high thyroglobulin levels at around 1 month of age and thus likely to develop hypothyroidism if iodine is not supplemented. The UIC levels improved only with enteral nutrition in ELGAN by 2 months of age. The trajectory of the earliest GA group, starting high, falling below other groups, and then rising, is clear. These babies are at risk for hypothyroxinemia in the first two months of life, which is a critical period for brain development.

Strengths of our study include its longitudinal design and collection and analysis of multiple samples. A major strength of our study is the inclusion of ELGAN near the limits of viability from 22 to 24 weeks gestation and follow up of their iodine and thyroid status till the time of their discharge home to delineate the time when they are most vulnerable. We could not monitor thyroid levels as frequently as urine iodine levels due to the limit on multiple blood sampling. Our study subjects were from an inner-city high-risk population with low income, low education and limited prenatal care; thus, the results of our study may specifically apply to other such communities across the US. 

## 7. Conclusions

We conclude that routine UIC monitoring during pregnancy should be considered and providing pregnant mothers with iodine containing prenatal vitamins should be standard-of-care [1,4,12]. ELGAN should receive TPN solutions supplemented with iodine to prevent potential hypothyroidism and prevent neurodevelopmental delays. 

## Figures and Tables

**Table 1:** Infants’ demographic characteristics.

Gestational Age (weeks)	Number (%)
22–27 28–32 33–36 37–40	13(26) 13(26) 14(28) 10(20)
**Birth weight (grams), Median (Q1–Q3)**	1690 (880–2551)
**Male Gender**	25(50)
**Race** Caucasian African American Hispanic Others	19(38) 25(50) 3(6) 3(6)
**Delivery** Vaginal Caesarian section	22(44) 28(56)
**Umbilical lines (Iodine prep)**	31(62)
**PICC lines** PICC line with Iodine PICC line with Chlorhexidine	17(34) 13(26) 4(8)

Data shown as Number (%) unless otherwise stated. PICC: Percutaneously inserted central line catheter.

**Table 2:** Maternal and Neonatal Clinical Characteristics.

Maternal Iodine Status & Thyroid Functions	Median (Q1, Q3)
Maternal Urine Iodine Concentration (mcg/L) Maternal iodine deficiency, N (%) Maternal Urine Iodine/Creatinine Ratio (mcg/g) Maternal Urine Creatinine Concentration (mg/dL) Maternal Free T4, (ng/dL) Maternal TSH, (µIU/mL)	98 (56, 177) 32 (64) 220 (117, 308) 47(27, 86) 0.484 (0.411, 0.540) 1.504 (0.860, 2.309)
**Prenatal Vitamin usage (%)**	44 (88)
**Breast Milk Iodine Concentration (mcg/L)**	71 (31, 281)
**Infant Iodine Status & Thyroid Functions:** TSH on ONBS (µIU/mL) Final TSH (40 weeks PMA or PTD) (µIU/mL) Final free T_4_ (40 weeks PMA or PTD) (ng/dL) Thyroglobulin (ng/mL) at 30–33 #DOL Urine Iodine Concentration (mcg/L) at 0–3 days (N = 48) Urine Iodine Concentration (mcg/L) at 7–10 days (N = 33) Urine Iodine Concentration (mcg/L) at 30–33 days (N = 25) Urine Iodine Concentration (mcg/L) at 60–63 days (N = 16) Urine Iodine Concentration (mcg/L) at 40 wks. PMA (N = 8) Urine Iodine Concentration (mcg/L) at PTD (N = 4)	7.2 (3.2, 11.5) 3.5 (1.9, 3.84) 1.26 (1.11, 1.48) 187.7 (156.5, 271.6) 151 (58, 2317) 103 (52, 213) 167 (54, 299) 213 (76, 440) 296 (193, 378) 315 (229, 525)
Total days on Parenteral nutrition	8 (0, 34)

Data shown as Median (Q1, Q3) unless otherwise stated. ONBS: Ohio newborn screen; PMA: Post menstrual age; PTD: Prior-to-discharge, DOL: Day of life.

**Table 3:** Comparison of Iodine levels according to Gestational age groups.

	22–27 Weeks *N* = 13	28–32 Weeks *N* = 13	33–36 Weeks *N* = 14	37–40 Weeks *N* = 10	*p*-Value
**Birth Weight (g)**	630 (600, 720)	1360 (1195, 1580)	2280 (1990, 2551)	3275 (3020, 3520)	
**Sex, No. (%)**					
**Male**	9(69)	8(62)	4(29)	4(40)	
**Female**	4(31)	5(38)	10(71)	6(60)	
**Race, No. (%)**					
**Caucasian**	2(15)	5(38)	5(36)	7(70)	
**AA**	10(77)	7(54)	8(57)	0(0)	
**Hispanic**	1(8)	0(0)	0(0)	2(20)	
**Other**	0(0)	1(8)	1(7)	1(10)	
**Maternal UIC (mcg/L)**	99 (56, 168)	63 (35, 154)	123 (81, 161)	146 (79, 277)	0.38 *
**Maternal Iodine deficiency,**					
**No (N(%))**	4(31)	4(31)	5(36)	5(50)	
**Yes (N(%))**	9(69)	9(69)	9(64)	5(50)	
**UIC (0–3 days) (mcg/L) ^a^**	2954 (2632, 3007) ^2,3,4^	362 (69, 985) ^1^	45 (25, 341) ^1^	96 (49, 105) ^1^	<0.001 *
**UIC (7–10 days) (mcg/L) ^a^**	56 (39, 98)	132 (67, 215)	119 (94, 294)	----	0.19 *
**UIC (30–33 days) (mcg/L) ^a^**	73 (34, 133) ^2^	299 (189, 457) ^1^	189 (113, 265)	----	0.017 *
**UIC (60–63 days) (mcg/L) ^a^**	146 (65, 336)	380 (230, 718)	----	----	0.090 *
**UIC (40 weeks PMA) (mcg/L) ^a^**	296 (193, 378)	----	----	----	
**UIC (PTD) (mcg/L) ^a^**	315 (229, 525)	----	----	----	

Data represented as N: Number (%), Median (Q1, Q3) unless stated otherwise. PMA: Post menstrual age, PTD: Prior-to-discharge. ^a^ Data not available for all subjects. *p*-values: * = Kruskal-Wallis test with Steel-Dwass multiple comparison adjustment for pairwise comparison, ^1^: Significantly different from 22–27 weeks; ^2^: Significantly different from 28–32 weeks; ^3^: Significantly different from 33–36 weeks; ^4^: Significantly different from 37–40 weeks.

## References

[1] Trumpff C., De Schepper J., Tafforeau J., Van Oyen H., Vanderfaeillie J., Vandevijvere S. (2013). Mild iodine deficiency in pregnancy in Europe and its consequences for cognitive and psychomotor development of children: A review. J. Trace Elem. Med. Boil..

[2] Zimmermann M. (2011). The role of iodine in human growth and development. Semin. Cell Dev. Boil..

[3] Zimmermann M. (2009). Iodine Deficiency. Endocr. Rev..

[4] Reuss M.L., Paneth N., Pinto-Martin J., Lorenz J.M., Susser M. (1996). The Relation of Transient Hypothyroxinemia in Preterm Infants to Neurologic Development at Two Years of Age. N. Engl. J. Med..

[5] Williams F.L., Watson J., Ogston S., Hume R., Willatts P., Visser T., the Scottish Preterm Thyroid Group (2012). Mild Maternal Thyroid Dysfunction at Delivery of Infants Born ≤34 Weeks and Neurodevelopmental Outcome at 5.5 Years. J. Clin. Endocrinol. Metab..

[6] Bath S., Steer C., Golding J., Emmett P., Rayman M.P. (2013). Effect of inadequate iodine status in UK pregnant women on cognitive outcomes in their children: Results from the Avon Longitudinal Study of Parents and Children (ALSPAC). Lancet.

[7] Vermiglio F., Presti V.P.L., Moleti M., Sidoti M., Tortorella G., Scaffidi G., Castagna M.G., Mattina F., Violi M.A., Crisà A. (2004). Attention Deficit and Hyperactivity Disorders in the Offspring of Mothers Exposed to Mild-Moderate Iodine Deficiency: A Possible Novel Iodine Deficiency Disorder in Developed Countries. J. Clin. Endocrinol. Metab..

[8] Bath S., Furmidge-Owen V.L., Redman C.W., Rayman M.P. (2015). Gestational changes in iodine status in a cohort study of pregnant women from the United Kingdom: Season as an effect modifier. Am. J. Clin. Nutr..

[9] Davis K., Li X., Adams-Huet B., Sandon L. (2017). Infant feeding practices and dietary consumption of US infants and toddlers: National Health and Nutrition Examination Survey (NHANES) 2003–2012. Public Health Nutr..

[10] Caldwell K.L., Pan Y., Mortensen M.E., Makhmudov A., Merrill L., Moye J. (2013). Iodine Status in Pregnant Women in the National Children’s Study and in U.S. Women (15–44 Years), National Health and Nutrition Examination Survey 2005–2010. Thyroid.

[11] Murray C.W., Egan S.K., Kim H., Beru N., Bolger P.M. (2008). US Food and Drug Administration’s Total Diet Study: Dietary intake of perchlorate and iodine. J. Expo. Sci. Environ. Epidemiol..

[12] Dasgupta P.K., Liu Y., Dyke J.V. (2008). Iodine Nutrition: Iodine Content of Iodized Salt in the United States. Environ. Sci. Technol..

[13] Ibrahim M., De Escobar G.M., Visser T.J., Duran S., Van Toor H., Strachan J., Williams F.L., Hume R. (2003). Iodine deficiency associated with parenteral nutrition in extreme preterm infants. Arch. Dis. Child. Fetal Neonatal Ed..

[14] Zimmermann M., Crill C.M. (2010). Iodine in Enteral and Parenteral Nutrition. Best Pr. Res. Clin. Endocrinol. Metab..

[15] Uhrmann S., Marks K.H., Maisels M.J., Friedman Z., Murray F., Kulin H.E., Kaplan M., Utiger R. (1978). Thyroid function in the preterm infant: A longitudinal assessment. J. Pediatr..

[16] Bodamer O.A., Leonard J.V., Halliday D. (1996). The relation between neonatal thyroxine levels and neurodevelopmental outcome at age 5 and 9 years in a national cohort of very preterm and/or very low birth weight infants. Pediatr. Res..

[17] Ares S., Escobar-Morreale H.F., Quero J., Durán S., Presas M.J., Herruzo R., De Escobar G.M. (1997). Neonatal Hypothyroxinemia: Effects of Iodine Intake and Premature Birth1. J. Clin. Endocrinol. Metab..

[18] Nath S.K., Moinier B., Thuillier F., Rongier M., Desjeux J.F. (1992). Urinary excretion of iodide and fluoride from supplemented food grade salt. Int. J. Vitam. Nutr. Res..

[19] Finch C.W. (2014). Review of Trace Mineral Requirements for Preterm Infants. Nutr. Clin. Pr..

[20] Agostoni C., Buonocore G., Carnielli V., De Curtis M., Darmaun D., Decsi T., Domellöf M., Embleton N., Fusch C., Genzel-Boroviczeny O. (2010). Enteral Nutrient Supply for Preterm Infants: Commentary From the European Society of Paediatric Gastroenterology, Hepatology and Nutrition Committee on Nutrition. J. Pediatr. Gastroenterol. Nutr..

[21] Belfort M.B., Pearce E.N., Braverman L.E., He X., Brown R.S. (2012). Low iodine content in the diets of hospitalized preterm infants. J. Clin. Endocrinol. Metab..

[22] Ghirri P., Lunardi S., Boldrini A. (2014). Iodine Supplementation in the Newborn. Nutrients.

[23] Domellöf M., Szitányi P., Simchowitz V., Franz A., Mimouni F., Braegger C., Bronsky J., Cai W., Campoy C., Carnielli V. (2018). ESPGHAN/ESPEN/ESPR/CSPEN guidelines on pediatric parenteral nutrition: Iron and trace minerals. Clin. Nutr..

[24] Cicalese M.P., Bruzzese E., Guarino A., Spagnuolo M.I. (2009). Requesting iodine supplementation in children on parenteral nutrition. Clin. Nutr..

[25] Clarridge K.E., Conway E.E., Bucuvalas J. (2013). Hypothyroidism and Iodine Deficiency in an Infant Requiring Total Parenteral Nutrition. J. Parenter. Enter. Nutr..

[26] Passos A.C.V., Barros F., Damiani D., Semer B., Cespedes W.C.J., Sannicola B., Tannuri A.C.A., Tannuri U. (2018). Hypothyroidism associated with short bowel syndrome in children: A report of six cases. Arch. Endocrinol. Metab..

[27] Ittermann T., Johner S., Below H., Leiterer M., Thamm M., Remer T., Völzke H. (2018). Interlaboratory variability of urinary iodine measurements. Clin. Chem. Lab. Med..

[28] Buyukgebiz A. (2013). Newborn screening for congenital hypothyroidism. J. Clin. Res. Pediatr. Endocrinol..

[29] Zimmermann M., Aeberli I., Andersson M., Assey V., Yorg J.A.J., Jooste P., Jukic T., Kartono D., Kusić Z., Pretell E. (2013). Thyroglobulin Is a Sensitive Measure of Both Deficient and Excess Iodine Intakes in Children and Indicates No Adverse Effects on Thyroid Function in the UIC Range of 100–299 μg/L: A UNICEF/ICCIDD Study Group Report. J. Clin. Endocrinol. Metab..

[30] WHO e-Library of Evidence for Nutrition Actions (eLENA). Iodine in pregnancy and lactation, Biological, behavioral and contextual rationale. https://www.who.int/elena/titles/bbc/iodine_pregnancy/en/.

[31] Greenwald I. (1955). Observations on the History of Goiter in Ohio and in West Virginia. J. Hist. Med. Allied Sci..

[32] Trumbo P., A Yates A., Schlicker S., Poos M. (2001). Dietary Reference Intakes. J. Am. Diet. Assoc..

[33] Berghout A., Wiersinga W. (1998). Thyroid size and thyroid function during pregnancy: An analysis. Eur. J. Endocrinol..

[34] Becker D.V., Braverman L.E., Delange F., Dunn J.T., Franklyn J.A., Hollowell J.G., Lamm S.H., Mitchell M.L., Pearce E., Robbins J. (2006). Iodine Supplementation for Pregnancy and Lactation—United States and Canada: Recommendations of the American Thyroid Association. Thyroid.

[35] Stagnaro-Green A., Abalovich M., Alexander E., Azizi F., Mestman J., Negro R., Nixon A., Pearce E.N., Soldin O.P., Sullivan S. (2011). Guidelines of the American Thyroid Association for the diagnosis and management of thyroid disease during pregnancy and postpartum. Thyroid.

[36] Patel A., Lee S.Y., Stagnaro-Green A., Mackay D., Wong A.W., Pearce E.N. (2019). Iodine Content of the Best-Selling United States Adult and Prenatal Multivitamin Preparations. Thyroid.

[37] Leung A.M., Pearce E.N., Braverman L.E. (2009). Iodine Content of Prenatal Multivitamins in the United States. N. Engl. J. Med..

[38] Teas J., Pino S., Critchley A.T., Braverman L.E. (2004). Variability of Iodine Content in Common Commercially Available Edible Seaweeds. Thyroid.

[39] Taylor P., E Okosieme O., Dayan C.M., Lazarus J.H. (2014). THERAPY OF ENDOCRINE DISEASE: Impact of iodine supplementation in mild-to-moderate iodine deficiency: Systematic review and meta-analysis. Eur. J. Endocrinol..

[40] Ares S., Quero J., De Escobar G.M. (2008). Iodine Balance, Iatrogenic Excess, and Thyroid Dysfunction in Premature Newborns. Semin. Perinatol..

[41] Leung A.M., Pearce E.N., Hamilton T., He X., Pino S., Merewood A., Braverman L.E. (2009). Colostrum iodine and perchlorate concentrations in Boston-area women: A cross-sectional study. Clin. Endocrinol..

[42] Ares S., Quero J., Duran S., Presas M.J., Herruzo R., De Escobar G.M. (1994). Iodine content of infant formulas and iodine intake of premature babies: High risk of iodine deficiency. Arch. Dis. Child. Fetal Neonatal Ed..

[43] Wolff J., Chaikoff I.L. (1948). The inhibitory action of excessive iodide upon the synthesis of diiodotyrosine and of thyroxine in the thyroid gland of the normal rat. Endocrinology.

